# Safety and Immunogenicity of Different Formulations of a Tetravalent Dengue Purified Inactivated Vaccine in Healthy Adults from Puerto Rico: Final Results after 3 Years of Follow-Up from a Randomized, Placebo-Controlled Phase I Study

**DOI:** 10.4269/ajtmh.19-0461

**Published:** 2020-03-02

**Authors:** Clemente Diaz, Michael Koren, Leyi Lin, Luis J. Martinez, Kenneth H. Eckels, Maribel Campos, Richard G. Jarman, Rafael De La Barrera, Edith Lepine, Irma Febo, David W. Vaughn, Todd M. Wilson, Robert M. Paris, Alexander C. Schmidt, Stephen J. Thomas

**Affiliations:** 1 University of Puerto Rico School of Medicine, San Juan, Puerto Rico;; 2 Walter Reed Army Institute of Research, Silver Spring, Maryland;; 3 GSK, Rockville, Maryland

## Abstract

Four formulations of an investigational tetravalent dengue purified inactivated vaccine, administered as two doses one month (M) apart, were previously shown to be immunogenic and well-tolerated up to M13 of the phase I study NCT01702857. Here, we report results of the follow-up from M14 to year (Y) 3. One hundred healthy Puerto Rican adults, predominantly dengue virus (DENV)–primed, were randomized 1:1:1:1:1 to receive placebo or vaccine formulations: 1 μg/serotype/dose adjuvanted with aluminum, AS01_E_ or AS03_B_, or aluminum-adjuvanted 4 μg/serotype/dose. No serious adverse events occurred. Two medically-attended potential immune-mediated disease cases, vaccination unrelated, were reported (groups 1 µg+Alum and 1 µg+AS03_B_). Of 14 instances of suspected dengue, none were laboratory confirmed. Geometric mean neutralizing antibody titers against DENV 1-4 waned from M14, but remained above pre-vaccination levels for DENV 1-3, with the highest values for group 1 µg+AS03_B_: 1220.1, 920.5, 819.4, and 940.5 (Y2), and 1329.3, 1169.2, 1219.8, and 718.9 (Y3). All formulations appeared to be safe and immunogenic during the 3-year follow-up.

## INTRODUCTION

Dengue is a vector-borne disease caused by the dengue viruses (DENVs) of the *Flavivirus* genus, with four serologically and genetically distinct serotypes (DENV-1, -2, -3, and -4). Although most DENV infections are mild or asymptomatic, symptomatic and severe dengue disease remains a major public health concern in tropical and subtropical regions, with a pronounced increase in incidence over the last 50 years.^1^ In Puerto Rico, dengue is endemic with suspected cases surpassing 10,000/year even in non-epidemic years.^2^

With no specific anti-DENV treatment available, prevention of dengue through immunization is considered an important approach to reduce the global burden. One live attenuated chimeric tetravalent vaccine (Dengvaxia, Sanofi Pasteur, Lyon, France) is currently licensed in 19 countries.^1,3^ However, the WHO recommends its administration in dengue-seropositive individuals,^1^ as an increased incidence of hospitalization and severe dengue was noted in dengue-seronegative vaccine recipients.^4^ Other investigational vaccines are under development.^5^

The immunogenicity and safety of an investigational tetravalent dengue purified inactivated vaccine (DPIV) formulated with different adjuvant systems have previously been reported.^6,7^ In a phase I clinical study conducted in a predominantly dengue-primed adult population in Puerto Rico, different formulations of 1 μg per DENV type 1–4 adjuvanted with alum, AS01_E_, or AS03_B_, or 4 μg per DENV type adjuvanted with alum were assessed. Three of the four formulations were shown to be highly immunogenic up to 12 months post-vaccination with two DPIV doses, and all were well tolerated.^8^ Here, we report final results from this trial after 3 years of follow-up.

## METHODS

This phase I, randomized, observer-blind, single-center study (NCT01702857) was conducted from October 2012 to January 2018 at the University of Puerto Rico Medical Sciences Campus, Puerto Rico. The study design has been described.^8^ In brief, participants were randomized 1:1:1:1:1 to receive two doses of different formulations of DPIV, administered 4 weeks apart: 1 μg/serotype/dose adjuvanted with aluminum (Group 1 μg+alum), AS01_E_ (Group 1 μg+AS01_E_) or AS03_B_ (Group 1 μg+AS03_B_), 4 μg/serotype/dose adjuvanted with aluminum (Group 4 μg+alum), or phosphate-buffered saline as placebo. Study participants were healthy adults aged 20–39 years, living in the Caribbean for more than 10 years. The inclusion/exclusion criteria were previously detailed.^8^

We previously reported results through month (M) 13.^8^ Here, we present the final study results for year (Y) 2 and Y3 of a 3-year follow-up, pertaining to secondary and exploratory objectives: to evaluate the safety of the four DPIV formulations from M14 to the study end, to assess exposure to DENV infection and occurrence of acute dengue illness throughout the study; and to evaluate the humoral immunogenicity of DPIV formulations to each of the four DENV types at various time points from M14 to Y3. Safety assessments were conducted for the total vaccinated cohort, comprising participants receiving at least one vaccine dose. Serious adverse events (SAEs), potential immune-mediated diseases (pIMDs), and medically attended adverse events (MAAEs) were collected by the investigator during scheduled visits or phone contacts conducted approximately every 4 months in Y2 and Y3, with serum samples collected every 4–8 months (Supplemental Figure 1).

All participants experiencing fever ≥ 38°C on two consecutive days were asked to contact the study staff on the second day. Following each report, the participant was invited for a visit involving a physical examination and a blood sample collection for virologic testing by reverse-transcriptase quantitative polymerase chain reaction (RT-qPCR).^9^ Suspected dengue was defined as fever (oral temperature ≥ 38°C) measured at least once on two consecutive days and no alternative diagnosis with reasonable certainty. Laboratory confirmation required RT-qPCR detection of DENV in blood samples. All cases of suspected and laboratory-confirmed dengue were described in detail.

Immunogenicity analyses were performed on the adapted according-to-protocol (ATP) cohort, including all evaluable participants who had data available for at least one immunogenicity end point at each time point (Supplemental Figure 1). Anti-DENV–neutralizing antibodies (NAbs) were measured using a micro-neutralization assay as previously described.^10^ Seropositivity was defined as a titer ≥ 10. Seropositivity rates (defined as the percentage of seropositive participants), geometric mean titers (GMTs), and tetravalent seropositivity rates were calculated. Geometric mean titers of NAb to each DENV type were calculated by time point and group with associated asymptotic 95% confidence intervals (CIs).

No prespecified statistical hypothesis testing was performed.

## RESULTS

Of the 100 vaccinated participants, 74 completed the study. Fourteen moved/migrated from the study area, 11 were lost to follow-up, and one was enrolled in a concurrent study. The ATP cohort at M14 included 79 participants; reasons for exclusion were essential serological data missing (*n* = 15), administration of vaccines/medication forbidden in the protocol (*n* = 3), and others (*n* = 3). Table 1 presents demographic characteristics of participants in the ATP cohort at Y3.

**Table 1:** Characteristics of participants (adapted according-to-protocol cohort for immunogenicity at M14)

	1 µg+alum (*N* = 16)	4 µg+alum (*N* = 16)	1 µg+AS01_E_ (*N* = 15)	1 µg+AS03_B_ (*N* = 16)	Placebo (*N* = 16)	Total (*N* = 79)
Age (years) at first vaccination, mean ± SD	26.9 ± 4.9	28.6 ± 5.7	30.1 ± 6.7	28.9 ± 6.2	28.9 ± 6.2	28.7 ± 5.9
Female, *n* (%)	11 (68.8)	12 (75.0)	8 (53.3)	6 (37.5)	13 (81.3)	50 (63.3)
American Hispanic/Latino ethnicity, *n* (%)	16 (100)	16 (100)	15 (100)	16 (100)	16 (100)	79 (100)
Tetravalent-positive participants at pre-vaccination, *n* (%)	12 (75.0)	14 (87.5)	12 (80.0)	15 (93.8)	13 (81.3)	66 (83.5)
Tetravalent-positive participants at M17–19, *n* (%)	14 (87.5)	15 (93.8)	14 (93.3)	16 (100)	13 (81.3)	72 (91.1)

M = month; M14 = 13 months post-dose 2; M17–19 = 16–18 months post-dose 2; *N* = number of participants in each group; *n* (%) = number (percentage) of participants in each category.

Safety data up to M13 have previously been published.^8^ From M14 to the study end, no SAEs were reported. Two pIMDs were recorded: one in the 1 μg+alum group (reactive arthritis, resolved by the study end) and one in the 1 μg+AS03_B_ group (rheumatoid arthritis, resolved with sequelae by the study end), with the onset at 883 and 581 days post–second vaccine dose, respectively. Both were medically attended, but were not considered as related to vaccination. No other MAAEs were reported.

Fourteen visits were conducted to evaluate suspected dengue illness for 9 participants: one in each of the 1 μg+alum and 1 μg+AS01_E_ groups, two in each of the 4 μg+alum and 1 μg+AS03_B_ groups, and three placebo recipients. None of the participants required hospitalization or intravenous fluids, and none of the cases evolved into an SAE. No laboratory-confirmed dengue cases were identified. For three participants (two in the placebo group and one in the 1 µg+AS01_E_ group), a ≥ 3-fold increase in NAb titers from pre- to post-visit for evaluation of suspected dengue was observed for at least one DENV type (Table 2). One of these participants had symptoms consistent with Zika virus illness during the Zika epidemic in Puerto Rico, but testing yielded negative results for Zika, dengue, and chikungunya viruses. Subsequent development of additional clinical signs and symptoms was indicative of an alternative diagnosis for these cases (Supplemental Table 1).

**Table 2:** Individual neutralizing antibody titers before and after suspected dengue illness for participants with suspected dengue

No.	Group	Blood sampling*	DENV-1	DENV-2	DENV-3	DENV-4
1	Placebo	M17–M19	30	5	5	198
		Y2	17	5	5	278
**2**	**Placebo**	**D28**	**198**	**151**	**75**	**639**
		**D56**	**110**	**232**	**143**	**2,247**
		M17–M19	126	35	38	581
		Y2	68	36	79	623
3	4 µg+alum	M7	38	81	35	56
		M10	42	65	5	34
4	4 µg+alum	M29–31	5,425	1,632	1,502	1,199
		Y3	3,347	1,106	2,367	723
5	1 µg+AS03_B_	M17–19	3,646	2,324	1,317	2,634
		Y2	5,762	2050	2074	3,764
**6**	**1 µg+AS01**_**E**_	**D0**	**48**	**12**	**645**	**5**
		**D7**	**233**	**110**	**3,345**	**286**
		**M4**	**332**	**91**	**1903**	**164**
		**M7**	**649**	**277**	**6,630**	**571**
		M10	342	131	2063	134
		M13	189	66	1,251	333
7	1 µg+alum	M17–19	600	547	460	664
		Y2	380	579	530	556
8	1 µg+AS03_B_	M13	2,430	704	713	1,288
		M17–19	1,523	184	319	104
**9**	**Placebo**	M13	4,944	1,286	746	693
		M17–M19	2,359	620	629	190
		**Y2**	**1730**	**686**	**916**	**285**
		**M29–31**	**5,032**	**1,110**	**1,013**	**197**

D = day; D28 = 1 month post-dose 1; D56 = 1 month post-dose 2; D0 = pre-vaccination; D7 = 7 days post-dose 1; DENV = dengue virus; M = month; M17–19 = 16–18 months post-dose 2; M7 = 6 months post-dose 2; M10 = 9 months post-dose 2; M29–31 = 28–30 months post-dose 2; Y3 = 36–38 months post-dose 2; M4 = 3 months post-dose 2; M13 = 12 months post-dose 2; Y = year; Y2 = 24–26 months post-dose 2. Bold values indicate the three participants with suspected dengue and a ≥ 3-fold rise in titers for at least one DENV serotype. Three participants had more than one suspect dengue visit.

* Closest time points before and after the suspected dengue visit.

**Figure 1.**
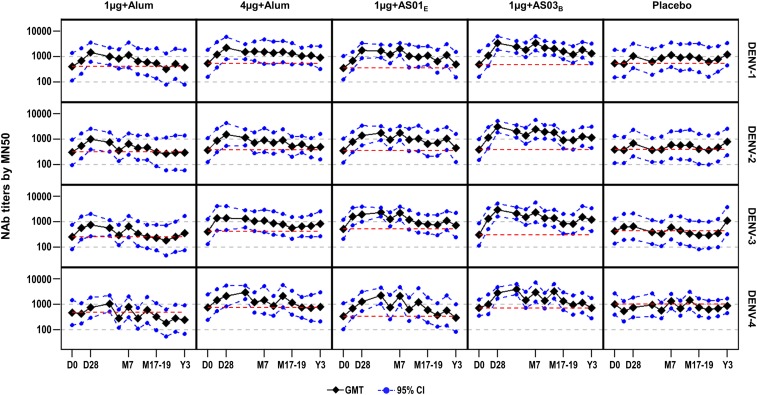
Persistence of neutralizing antibody responses up to Y3 post-vaccination (adapted according-to-protocol cohort for immunogenicity). D = day; DENV = dengue virus; D0 = pre-vaccination; D28 = 1 month post-dose 1; GMT = geometric mean titer; LL = lower limit; MN50 = micro-neutralization assay; M = month; M7 = 6 months post-dose 2; M17–19 = 16–18 months post-dose 2; NAb = neutralizing antibody; UL = upper limit; Y = year; Y3 = 36–38 months post-dose 2. The red dotted line represents pre-vaccination values. GMTs were computed by taking the antilog of the mean of the log_10_ titer transformations. Antibody titers below the cutoff of the assay were given an arbitrary value of half the cutoff (5).

NAb GMTs against DENV types 1–4 at Y2 and Y3 post-vaccination waned slightly from M14 levels, but remained higher than pre-vaccination levels across investigational groups, except for DENV-4. The highest GMTs were observed in the 1 µg+AS03_B_ group: 1220.1, 920.5, 819.4, and 940.5 at Y2; 1329.3, 1169.2, 1219.8, and 718.9 at Y3 for DENV types 1–4, respectively. Increased GMTs were observed at Y3 post-vaccination for placebo recipients (1222.6 [DENV-1], 780.2 [DENV-2], 1115.5 [DENV-3], and 871.6 [DENV-4]) (Figure 1). NAb seropositivity rates decreased from M14 to Y2, increased slightly at the M29–31 time point, and then declined at Y3. Seropositivity rates at Y3 ranged from 92.3% to 100% for any of the four DENV types, in all groups except the 1 µg+alum group (78.6–85.7%). The percentages of tetravalent responders at Y3 were 78.6% (95% CI: 49.2–95.3) in the 1 µg+alum group, 92.9% (95% CI: 66.1–99.8) in the 4 µg+alum group, 92.3% (95% CI: 64.0–99.8) in the 1 µg+AS01_E_ group, 100% (95% CI: 73.5–100) in the 1 µg+AS03_B_, and 93.8 (95% CI: 69.8–99.8) in participants receiving placebo. Fold changes in NAb titers for each DENV type are presented in Supplemental Figure 2.

## DISCUSSION

The final results of this phase I trial of the DPIV investigational vaccine in a highly primed, dengue-endemic population extend previous findings indicating that all four evaluated formulations were well tolerated and immunogenic, with no safety concerns identified over 3 years from vaccination. Of note, this trial was not designed to explore efficacy or evaluate any differences between dengue-primed and unprimed individuals following DPIV administration.

The safety results are consistent with previous observations for the tetravalent DPIV in a first-time-in-human trial in healthy, dengue-naive adults aged 18–39 years, with all formulations being well tolerated and no SAEs observed up to 12 months post-vaccination with a two-dose series.^7^ Similarly, a monovalent aluminum-adjuvanted vaccine containing the same DENV-1 antigen as DPIV showed an acceptable safety profile following administration of different antigen amounts according to a two-dose schedule in healthy adults.^6^ In this small phase 1 study, no laboratory-confirmed dengue cases were identified during the three years of follow-up. Notably, the majority (90%) of the participants had evidence of prior DENV infection at vaccination, which limits the generalization of these results to other populations.

Consistent with previous immunogenicity data at M13,^8^ the 1 µg+alum appeared less immunogenic than the other three formulations, with the 1 µg+AS03_B_ showing the highest persistence of NAb at Y3 following vaccination. At all follow-up time points, observed GMTs against DENV types 1–4 were higher for the 1 µg+AS03_B_ formulation, and all participants receiving 1 µg+AS03_B_ had a tetravalent response and a positive fold change at Y3. However, these data are difficult to interpret in the absence of a correlate of protection against dengue. Post-vaccination NAbs, measured by the 50% plaque reduction neutralization test, elicited by the licensed tetravalent vaccine were previously associated with vaccine efficacy against dengue.^11^ Although this comparison is limited by the difference in assays used, GMTs across all DENV types observed in our study indicate that increased individual antibody levels persist up to Y3.

An increase in NAb GMTs and seropositivity rates was observed during the last year of the follow-up, in all groups, including placebo recipients. Asymptomatic exposure to DENV or cross-reactive *Flavivirus* during this period may be an explanation. Of note, during the study period, DENV-1 and DENV-4 were the main circulating serotypes in Puerto Rico.^12^ Although none of the study participants was clinically diagnosed with a Zika virus infection, Y3 coincided with a Zika virus epidemic in Puerto Rico in 2016.^13^ Cross-reactivity between antibodies to Zika virus and DENV has previously been reported^14,15^ and is likely due to substantial similarity in the sequence type of surface glycoprotein E.^16^

Based on these results, the 1 µg+AS03_B_ formulation administered according to different dose schedules was assessed in a subsequent clinical trial (NCT02421367; L. Lin et al., unpublished data). Subsequently, the development of DPIV as a homologous prime-boost vaccine candidate was not pursued any further, but a heterologous prime-boost schedule using DPIV in combination with a live attenuated dengue vaccine is currently under evaluation.

## CONCLUSION

Vaccination with DPIV appeared safe and immunogenic over 3 years after vaccination in an adult, dengue-endemic population.

##  Supplemental figures and table 

Supplemental materials
