# Supplementary material for: Safety and Immunogenicity of Different Formulations of a Tetravalent Dengue Purified Inactivated Vaccine in Healthy Adults from Puerto Rico: Final Results after 3 Years of Follow-Up from a Randomized, Placebo-Controlled Phase I Study
Source: Am J Trop Med Hyg. 2020 Mar 2;102(5):951–4. doi: 10.4269/ajtmh.19-0461 (PMC7204593; doi:10.4269/ajtmh.19-0461)
Supplement: Supplementary file 1 [file tpmd190461.SD1.pdf]

Supplemental Figure: Study design

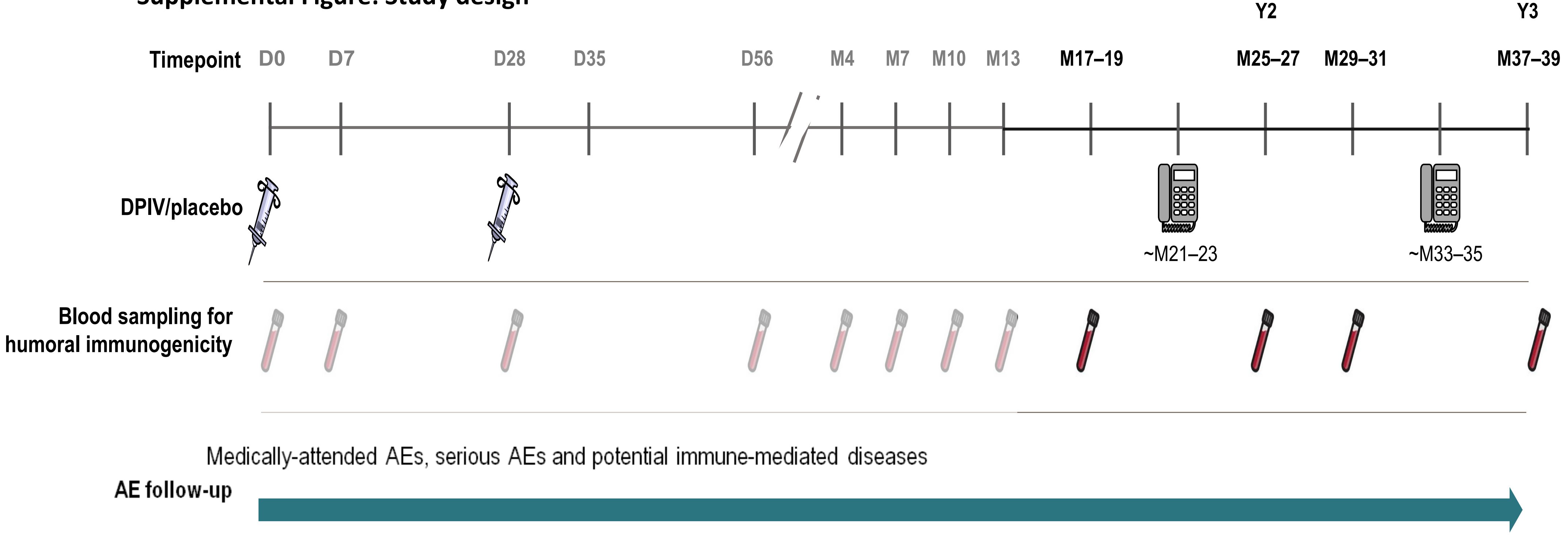

D, day; M, month; Y, year; DPIV, investigational tetravalent dengue purified inactivated vaccine; AE, adverse event.
Note: Outcomes for greyed-out timepoints were previously reported.8
Clinic visits/phone contacts corresponded to timepoints pre- (M17-19, M29-31), during (M21–23, M33–35) and post- (M25–27, M37–39) dengue season.

Supplemental Figure: Fold increase in neutralizing antibody titers between prevaccination and M14 (A) M14 and Y3 (B), and pre-vaccination and Y3 (C) in primed participants (adapted according-to-protocol cohort for immunogenicity)

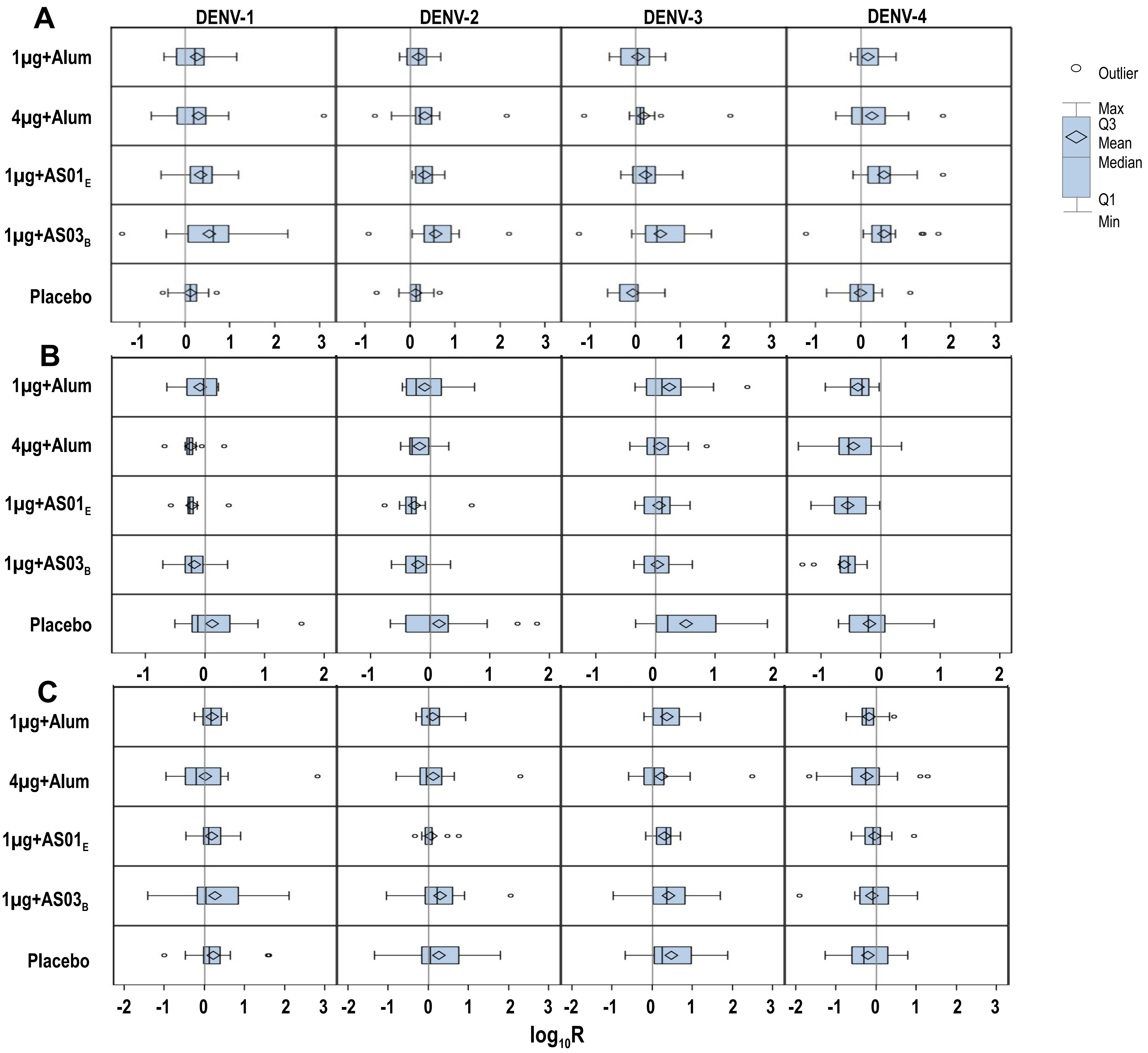

M, month; Y, year; DENV, dengue virus; M14, 13 months post-dose 2; Y3, 36–38 months post-dose 2.
